# Societal influence and psychological distress among Indonesian adults in Java on the early Omicron wave of COVID-19

**DOI:** 10.2144/fsoa-2023-0104

**Published:** 2023-08-29

**Authors:** Annette d'Arqom, Muh Akram, Nafesa Shafira Azzahranisa, Mhd Zamal Nasution, Endang Retno Surjaningrum, Junaidah Yusof

**Affiliations:** 1 Division of Pharmacology & Therapy, Department of Anatomy, Histology, & Pharmacology, Faculty of Medicine, Universitas Airlangga, 60131, Indonesia; 2 Airlangga Research Group for Translational Medicine & Therapeutic, Universitas Airlangga, 60131, Indonesia; 3 Faculty of Medicine, Universitas Airlangga, Surabaya, 60131, Indonesia; 4 Postgraduate School, Universitas Airlangga, Surabaya, 60286, Indonesia; 5 Faculty of Psychology, Universitas Airlangga, Surabaya, 60286, Indonesia; 6 School of Human Resource Development & Psychology, Faculty of Social Sciences & Humanities, Universiti Teknologi Malaysia, Johor Bahru, 81310, Malaysia

**Keywords:** anxiety, desirability, distance, information, mental health

## Abstract

**Aim:**

Changes during the COVID-19 pandemic might create pressure on different people, thus this study aimed to measure respondents' psychological distress during the early phase of the Omicron wave in Java Island.

**Methods:**

A web-based survey on societal influence and COVID-19-related psychological distress was distributed through social media in November–December 2021, and received 396 responses.

**Results:**

This study showed that almost 50% of respondents faced psychological distress during the early phase of the Omicron variant, especially concerning hypervigilance and avoidance. Several sociodemographic factors might contribute to the incidence of psychological distress including comorbidity, age and education.

**Conclusion:**

Taken together, the incidence of COVID-19-related psychological distress was still found in the early phase of the Omicron variant, especially among young adults.

## Plain language summary

The COVID-19 pandemic hits every aspect of human life, including the social and psychological aspects. This study was conducted to measure these changes in Indonesian adults who stayed on Java Island during the early phase of the Omicron wave. Using an online questionnaire, 396 responses were received and analyzed. The results showed that almost half of the respondents still had symptoms of psychological distress. Several sociodemographic factors might contribute to the incidence of psychological distress including comorbidity, age, and education. Understanding these factors might bring benefits for future pandemic preparedness.

## Tweetable abstract

Almost half of 396 Indonesian adults in Java faced psychological distress during early phase of the Omicron wave of the COVID-19 pandemic.

## Graphical abstract

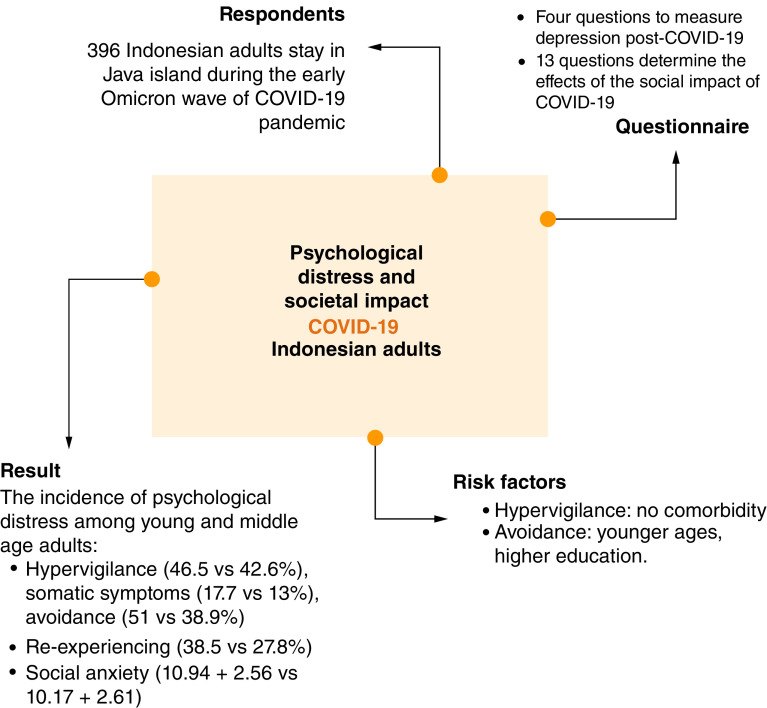

The COVID-19 pandemic, the biggest pandemic of the 21st history, forced all aspects of human life to change. The exact origin of SARS-CoV-2 is not yet definitively confirmed, but it is believed to have originated in bats and possibly transmitted to humans through an intermediate animal host. One of the earliest known cases emerged in a seafood market in Wuhan, China, where wild animals were sold, suggesting a possible zoonotic origin [1]. COVID-19 primarily spreads through respiratory droplets produced when an infected person coughs, sneezes, talks or breathes. The virus can also spread by touching surfaces or objects contaminated with the virus and then touching the face, especially the eyes, nose or mouth [2]. The diagnosis of COVID-19 involves several methods, including molecular tests like quantitative real-time polymerase chain reaction (qRT-PCR) and antigen tests, as well as serological tests (antibody tests) [3]. In May 2023, the WHO decided to revoke the pandemic status after decreasing in the mortality of COVID-19 cases. For more than 3 years of the pandemic, COVID-19 infects more than 765 million people, with a fatality of 6.9 million cases [4]. As of the end of the COVID-19 pandemic, the number of COVID-19 cases in Indonesia totalled 6,048,204, with 156,371 deaths [5].

The uncertainties and fears associated with the virus outbreak, mass lockdowns and economic recession causing psychological distress and mental health problems globally. A review involving 19 studies of the general population in China, Spain, Italy, Iran, the USA, Turkey, Nepal and Denmark in 2020, found several mental health disorders, such as anxiety, depression, post-traumatic stress disorder, psychological distress and stress in various incidences [6]. In Indonesia, 20–25% of people suffered anxiety symptoms, as well as depression and stress during the first and second wave of the COVID-19 pandemic [7–9]. People are significantly affected by these mental health effects as a result of psychological stressors related to the COVID-19 pandemic because loneliness, worry and depression further impair the immune system [10]. Many people were affected directly and indirectly due to SARS-CoV-2 infection and it might not have resolved within a short period of time.

The social impacts of COVID-19 could be divided into several aspects including social distancing, social anxiety, social information, social desirability and social adaptation. As social distancing was necessary to limit the spreading of SARS-CoV-2, anxiety symptoms correlated to limited social interaction, social isolation and quarantine [11]. Social anxiety due to COVID-19 has been reported widely [12]. Interestingly, a study in 386,432 students in China found that face mask knowledge and usage reduced the incidence of anxiety [13]. Increasing demand for information from many sources, including social media might increase COVID-19 knowledge and affect individual behaviors related to COVID-19 transmission; however, much unclear information spreading also might induce mental health problems [14]. With the prolonged pandemic, the ability to adapt became important to protect someone from transmission [15]. Additionally, social desirability might influence someone's behaviors including compliance with the government's strategies [16].

A higher impact of the pandemic was felt by the most vulnerable groups, such as youth, the elderly, low-income individuals, people with comorbidity, etc. Moreover, with the application of social distancing, decreasing in social activity and interaction, losing family members, losing a job and other stressful events caused mental health problems [9]. A study of 381 Italian COVID-19 patients found 30.2% of the respondents experienced post-traumatic stress disorder (PTSD) and 17.3% of them experiencing depressive disorder [17]. Mental health problems such as depression, anxiety, stress and post-traumatic stress disorders also reported in various countries, including USA, Poland, China, Hongkong, Thailand, Philippines, Vietnam, etc. [18–21]. Several sociodemographic factors, such as age, sex, marital status, pre-existing diseases, employment and having family, contributed to mental health problems. A study in the USA showed that older people were more likely to adapt to changes caused by COVID-19; however, the severity and mortality of COVID-19 was higher among older people [18]. *Vice versa*, a study in seven Asian countries concluded that younger age was more likely to develop mental health problems [20]. Sex also contributed to mental health disorders development where female has a higher risk [19,20]. Individuals with stable income or having work during the pandemic were less likely to have anxiety, depression or stress symptoms [22]. Loneliness correlated to a higher incidence of mental health issues, thus married individuals or individuals with children showed lower odds to develop these problems [20]. Interestingly, migrant workers and students have higher odds of developing anxiety, depression or post-traumatic stress disorders [19]. People with higher education levels, believe the health professional and less exposure to disinformation were less likely to have the symptoms. In addition, contact history of COVID-19 infection, being chronically ill, and having pre-existing mood disorders contributed to the development of mental health problems during the pandemic [6,19,20,23].

Unfortunately, patients might develop sequelae or new symptoms following COVID-19 recovery. Seriously ill COVID-19 patients and unvaccinated people might get long-COVID symptoms that might involve the cardio and cerebrovascular system such as tachycardia and coagulation, an endocrine system such as Type 2 diabetes, myalgia or fatigue syndrome, neurologic systems such as neurocognitive dysfunction, etc [24]. A review involving eight studies during the first one and half years of the COVID-19 pandemic showed 11–28% depressive disorders among COVID-19 survivors 12 weeks post COVID-19 infection. This incidence was not related to COVID-19 severity [25]. Moreover, fatigue and cognitive impairment were also observed to be persistent for more than 12 weeks after the infection [26].

Even though the incidence of mental health problems varies between countries, the incidence is more likely to be higher in developing countries, due to the feeling of unpreparedness with the lack of appropriate medical facilities and equipment [21]. Vaccine hesitancy and availability also play a major role in the high mortality during the second wave of the COVID-19 pandemic [27]. At the beginning of the COVID-19 vaccination program in Indonesia, the acceptance rate is 64.8% and increased to more than 90% during the Delta wave COVID-19 pandemic [28]. The governments' strategies to reduce SARS-CoV-2 transmission, such as lockdowns, strict regulation of transportation and wearing masks, vaccination program, etc. bring benefits to reduce the morbidity and mortality rate of COVID-19 cases. Those strategies also significantly lower the prevalence of clinically significant depressive symptoms [29], as reported in a study involving more than 20,000 UK adults which concludes that COVID-19 vaccination alleviates COVID-19 related psychological distress [30]. Thus, understanding the COVID-19 psychological distress, the societal impact of the pandemic, and its determinant factors are vital for a better understanding of the vulnerability's variations during the COVID-19 outbreak, thus can be beneficial to deal with future catastrophic events, especially in a diverse country like Indonesia.

## Methods

### Ethics, study design & data collection

This cross-sectional study was held from November to December 2021 and approved by the Health Research Ethics Committee. The survey was conducted using a survey generator (www.surveyplanet.com) and distributed online using social media and email. A landing page was provided before entering the survey providing an explanation of the aims of the study, informed consent and respondents could agree to publish an anonymous response. The consent was given by clicking the YES option, and multiple submissions were prevented as in Checklist for Reporting Results of Internet E-Surveys CHERRIES guidelines. This study followed the Declaration of Helsinki and the Strengthening the Reporting of Observational Studies in Epidemiology (STROBE) guidelines. The inclusion criteria were Indonesian over 18 years old and stayed in Java Island during the early Omicron COVID-19 pandemic. The exclusion criterion was incomplete submission. To have an accurate finding and preventing the risk of reporting false-negative or false-positive findings, sample size was calculated with 5% margin of error, 95% confidence level and 100 million for population size. Using a sample size calculator (http://www.raosoft.com/samplesize.html), the minimum sample required for this study was 385 respondents.

### Survey instrument

A set of questionnaires covered respondents' basic sociodemographic information, four questions to measure depression post-COVID-19 and 13 questions of the Societal Influences Survey Questionnaire (SISQ) to determine the effects of the social impact of COVID-19. Four questions were asked to measure how respondents' experienced hypervigilance, somatic symptoms, avoidance and re-experience of COVID-19 using the disaster-related psychological screening test (DRPST) during the past month. The items were measured using a five-point Likert scale (not at all (1) to extreme (5)) [31]. Thirteen out of fifteen questions of the SISQ were used to measure social distance, social anxiety, social desirability and social information during the COVID-19 pandemic. The items were measured using a four-point Likert scale (never (1) to often (4) [32]. Two questions of SISQ on social adaptation were omitted from the questionnaire due to low reliability (<0.5). The questionnaire was translated by one medical doctor, two psychologists and one social science expert prior to face validity in 20 respondents to measure respondents' level of understanding of the questions, including language, sentence and format of the question.

### Analytical procedure

Respondents were divided into two groups based on their age, namely, young adult (18 to 25 years old) and middle age adult (>25 to 55 years old). The score of the DRPST was calculated using scores ranging from 4 to 20. Higher scores indicated more severe depression. Moreover, the sum of each SISQ categorized item was calculated. Higher scores indicated the respondents maintained greater distance, obtained more informative, experienced more anxiety, and adapt more to COVID-19. The reliability value was generated as a part of the pilot test. Pilot test was conducted as it is a crucial step before the actual data collection since it allows to determine whether the instrument will work successfully on a smaller scale before using it in a larger study. The pilot test for this study shows great reliability and good internal consistency of Cronbach's Alpha value. The reliability measurement on the survey data showed a Cronbach Alpha coefficient of 0.799 for DRPST, 0.649 for Social Distance, 0.657 for Social Anxiety, 0.649 for Social Desirability and 0.937 for Social Information. The Mann-Whitney U test was used to compare mean depression and each societal influential component between young adult and middle age adult groups. To investigate the predictive factors of each symptom of depression, binary logistic regression was used. To perform this analysis, a Likert scale of DRPST was contracted to 0 = not experienced the symptom, and 1 = experienced the symptom. Significant predictive factors were defined as variables with p < 0.05.

## Results

### Characteristics of respondents

Three hundred ninety-six valid and completed responses were analysed. More than 70% of respondents were young adults (72.72%) and 67.42% were females. Ratio between high school graduates and higher-level graduates was almost 1 to 1. Only 20% of respondent were ever diagnosed with COVID-19 and 17.42% presented a comorbidity. Almost 77% of respondents reported that their self-claimed economic status compared with their neighbors was average. Characteristics of respondents are summarized in Table 1.

**Table 1.** Characteristics of respondents.

Sociodemographic factor	Young adult (n = 288)	Middle age adult (n = 108)
Sex		
Male	77 (26.7%)	52 (48.1%)
Female	211 (73.3%)	56 (51.9%)
Education		
High school graduate	208 (72.2%)	4 (3.7%)
University graduate	80 (27.8%)	104 (96.3%)
History of COVID		
Never diagnosed	233 (80.9%)	83 (76.9%)
Diagnosed	55 (19.1%)	25 (23.1%)
Comorbidity		
Does not have	250 (86.8%)	77 (71.3%)
Have comorbidity	38 (13.2%)	31 (28.7%)
Economy status		
Average and below	220 (76.4%)	82 (75.9%)
Above average	68 (23.6%)	26 (24.1%)
Supplement consumption		
Consumed	219 (76%)	86 (79.6%)
Not consumed	69 (24%)	22 (20.4%)

### COVID-19 related psychological distress & its determinant factors

**Figure 1.**
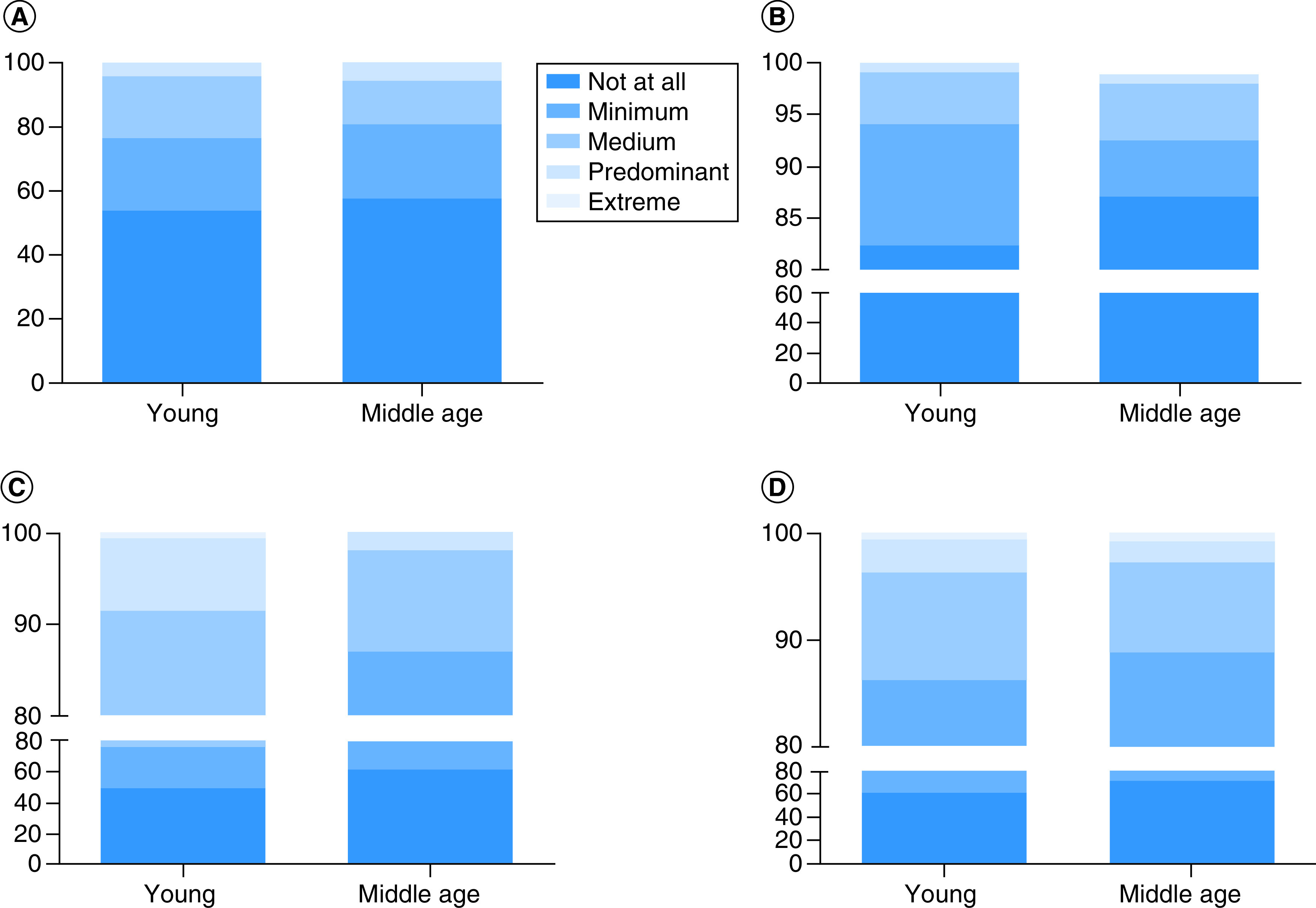
Incidence of COVID-19-related psychological distress among young and middle age adults in Java Island, Indonesia during early Omicron wave. **(A)** Hypervigilance. **(B)** Somatic symptom. **(C)** Avoidance. **(D)** Re-experience.

Four questions of the DRPST questionnaire screened major depressive disorder or post-traumatic stress disorder (DRPST) after a disaster. This study showed that during the early wave of the Omicron variant, no significant differences were noted of the incidence of psychological distress among young and middle age adults including hypervigilance (46.5 vs 42.6%), somatic symptoms (17.7 vs 13%). While the incidence of avoidance (51 vs 38.9%, p = 0.031) and re-experiencing (38.5 vs 27.8%, p = 0.047) were significantly higher in young adults (Figures 1A to D). However, using a 5 point-Likert-scale (1 to 5), the results showed a significant difference between mean total score from young adults 6.42 ± 2.42 and middle age adults 5.85 ± 2.37 (p = 0.009) on total score of DRPST. Significant differences between young and middle age adults were also observed in avoidance (1.85 ± 1.01 vs 1.54 ± 0.77, p = 0.008). However, no significant differences were noted concerning hypervigilance (1.75 ± 0.92 vs 1.68 ± 0.92, p = 0.432), somatic symptoms (1.25 ± 0.59 vs 1.21 ± 0.59, p = 0.315) and re-experience (1.57 ± 0.85 vs 1.43 ± 0.80, p = 0.061).

**Figure 2.**
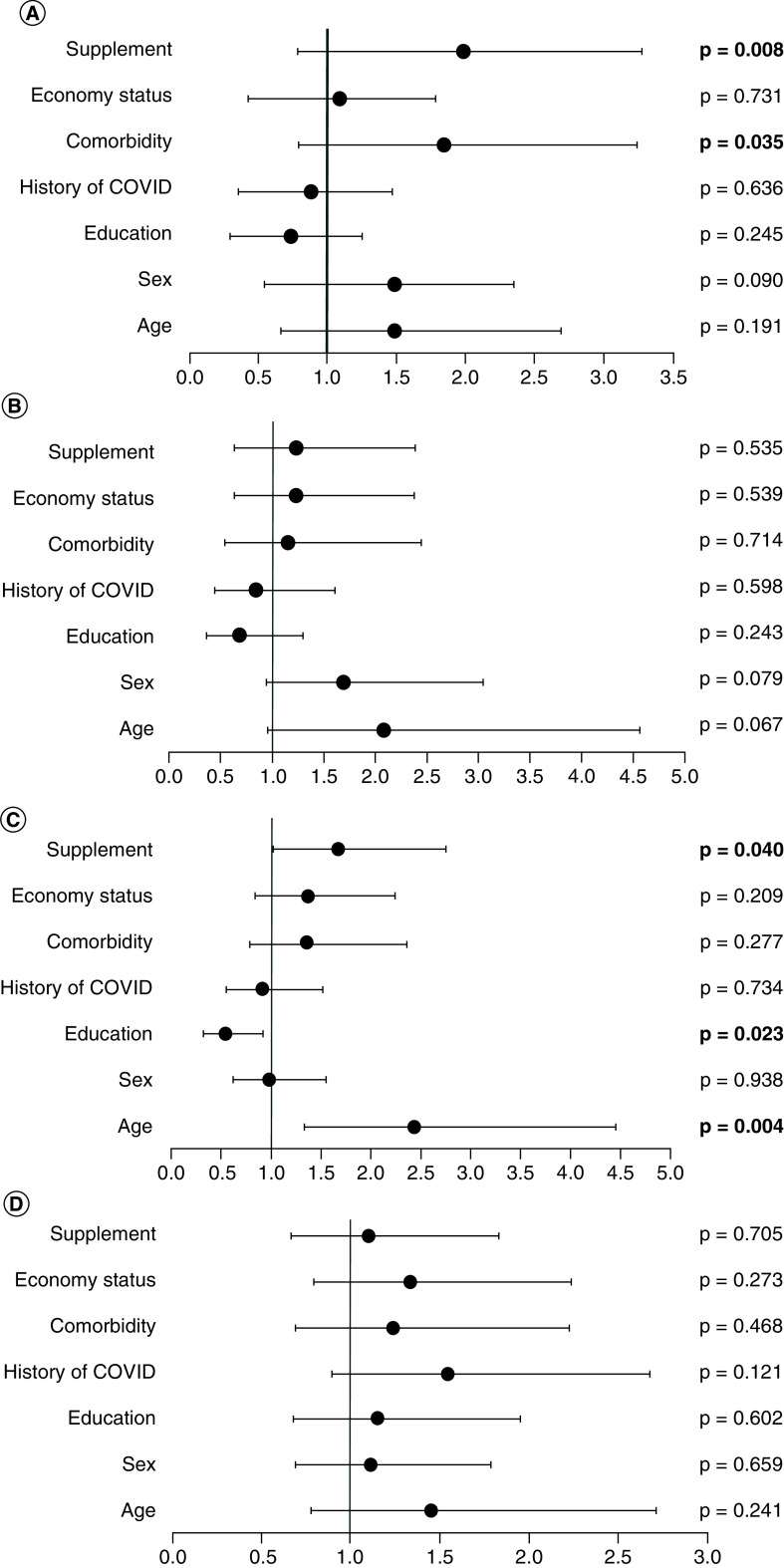
Forest plot of predictive factors on each distress symptom. **(A)** Hypervigilance. **(B)** Somatic symptom. **(C)** Avoidance. **(D)** Re-experience. Boldface numbers indicates significant p-value.

Moreover, to predict sociodemographic factors associated with respondents' COVID-19 related psychological distress, binary logistic regression was performed. Figure 2 shows the results from modelling of the outcome as a function of several independent variables including age, sex, education level, history of COVID-19, comorbidity, economic status and supplement consumption. Each component of the four symptoms of DRPST was categorized into two categories (not experiencing and experiencing). The results showed that respondents without comorbidity conditions were 1.842-times more likely to have hypervigilance (95% CI 1.045 to 3.244, p = 0.035), while respondents that consumed supplements were 1.983 more likely to experience hypervigilance (95% CI 1.2 to 3.277, p = 0.008) and 1.675-times more likely to experience avoidance (95% CI 1.023 to 2.744, p = 0.040). Moreover, respondents at younger ages were more likely to have avoidance symptoms as they were 2.439-times more likely to have avoidance symptoms (95% CI 1.339 to 4.443, p = 0.004). In addition, higher education was a negative predictor for avoidance symptoms, as respondents who graduated from high school were 0.546 less likely to have this symptom compared with respondents with a diploma or bachelor's degree (95% CI 0.323 to 0.921, p = 0.023). Our results showed that sociodemographic factors were not significantly related to somatic symptoms and re-experiencing symptoms from COVID-19 related psychological distress (Figures 2A to D).

### Societal influential

Using SISQ, this study showed a total score of SISQ (37.83 ± 5.73 vs 36.30 ± 5.95, p = 0.018) and social anxiety (10.94 ± 2.56 vs 10.17 ± 2.61, p = 0.011) significantly differed between young and middle age adults. However, this study did not find any differences concerning social distancing (10.89 ± 3.04 vs 10.44 ± 3.06, p = 0.167), social desirability (10.71 ± 1.36 vs 10.59 ± 1.62 p = 0.933) and social information (5.29 ± 1.61 vs 5.09 ± 1.77, p = 0.302) between two age groups. Furthermore, no significant differences were found concerning the total SISQ and each component of societal influence when analyzed based on the sociodemographic factors (Table 2). Social anxiety showed anxiety symptoms that correlated to limited social interaction, social isolation and quarantine [11].

**Table 2.** Mann-Whitney U of societal influence.

Sociodemographic factors	n	Social distance		Social anxiety		Social desirability		Social information		Total SISQ	
		z	p-value	z	p-value	z	p-value	z	p-value	z	p-value
Age		-1.383	0.167	-2.545	**0.011**	-0.084	0.933	-1.033	0.302	-2.373	**0.018**
Young adults	288										
Middle age adults	108										
Sex		-0.904	0.366	-0.982	0.326	-0.170	0.865	-0.205	0.838	-0.966	0.334
Male	129										
Female	267										
Education		-0.887	0.375	-0.658	0.511	-0.074	0.941	-0.233	0.816	-1.009	0.313
High school graduate	212										
University graduate	184										
History of COVID		-0.174	0.862	-0.314	0.753	-1.102	0.271	-1.590	0.112	-0.021	0.983
Never diagnosed	316										
Ever diagnosed	80										
Comorbidity		-0.254	0.799	-0.592	0.554	-0.056	0.955	-1.239	0.215	-0.021	0.983
Does not have	327										
Have comorbidity	69										
Economic status		-0.029	0.977	-1.576	0.115	-0.318	0.751	-0.008	0.994	-0.350	0.726
Average and below	302										
Above average	94										
Supplement		-0.518	0.605	-0.847	0.397	-0.007	0.994	-0.228	0.820	-0.092	0.926
Consumed	305										
Not Consumed	91										

Boldface numbers indicates significant p-value.

SISQ: Societal Influences Survey Questionnaire.

## Discussion

Indonesia is the fourth most populated country in the world with 281,216,462 people [33]. Fifty-six percent of them reside in Java Island and make it the densest populated island in Indonesia. Because the capital of Indonesia is located on this island, the infrastructures in Java are the most developed compared with other islands [34]. However, even though Java is the most developed island, poverty remains a challenge. This inequality leads to disparities in many sectors including health, between each region in Indonesia including in Java itself [35]. With this characteristic, it make sense that almost one-half of Indonesian COVID-19 cases were reported from Java Island [36].

The COVID-19 pandemic greatly impacts health, economics, social issues, education, politics and human relationships. City lockdowns or high-scale social restrictions change and limit human habits and activities [37]. Domestic violence reportedly increased during this period [38]. Increasing chores including distance learning, has been associated with depression, anxiety, and stress both within the family unit and among students [39,40]. Overload and chaotic work during the outbreak caused burnout syndrome, such as among health workers, teacher, and students, which is related to psychological distress [41–43]. Losing family members or experiencing COVID-19 itself might lead to psychological distress, anxiety, phobia, obsessive compulsive disorders, etc. [44]. For COVID-19 survivors, sequelae or a few problems might follow the recovery. Several symptoms were including persistent fatigue and cognitive impairment, which might related with neurological problems [24,26].

The incidence of depression, anxiety and stress was reported in 25% of 610 Indonesian mothers with school-age children [8]. This study showed that during the early wave of the Omicron variant, Indonesian adults in Java still faced COVID-19 related psychological distress, shown in symptoms of hypervigilance (46.5 vs 42.6%), somatic symptoms (17.7 vs 13%), avoidance (51 vs 38.9%) and re-experiencing (38.5 vs 27.8%) among young and middle age adults, respectively. Less than 50% of respondents experienced distress, that might have been caused by a high rate of vaccination coverage after the Delta wave. As of 19 November 2022, Indonesia vaccination coverage was 75.98 and 63.74%, for the first dose and second dose, respectively [45]. A study involving 910 Indonesian adults showed that younger ages, having better knowledge on vaccination, and having medical background were more likely to accept the COVID-19 vaccination program [28]. COVID-19 vaccination program also concluded to reduce distress including fear of infection, hospitalization and mortality due to COVID-19 as reported in 8090 US adults [46]. Furthermore, because Omicron occurred in the third year of the COVID-19 pandemic, the general population tended to show better resilience to the pressure compared with the early pandemic, as reviewed by Manchia *et al.* [47]. One study among 1,305 patients positive for Omicron in China also supported this finding, as the prevalence of depression, anxiety and perceived stress in this population was lower than during the early pandemic (9.03, 4.60 and 17.03%, respectively) [48].

Several sociodemographic factors might be related to each distress symptom. This study found that respondents without comorbidities were 1.842-times more likely to have hypervigilance (95% CI 1.045 to 3.244, p = 0.035) or continuously assess the threat situation. Incidence of hypervigilance and depression was more likely related to COVID-19 compared with hypervigilance with anxiety, as reported globally among 7034 adults [49]. This finding contrasted with the result among 228,367 patients with COVID-19 positive test results in USA during the early pandemic, as 25.6% presented posttraumatic stress disorder (PTSD), associated with cardiovascular diseases. This study also reported that PTSD might increase the risk of hospitalization and mortality, even though the incidence was higher among patients with other psychiatric conditions other than PTSD such as depression, anxiety, alcohol disorder, bipolar disorder, etc [50].

Hypervigilance and avoidance also correlated with respondents consuming dietary supplements, as 1.983 were more likely to experience hypervigilance (95% CI 1.2 to 3.277, p = 0.008) and 1.675-times more likely to experience avoidance (95% CI 1.023 to 2.744, p = 0.040). Several nutrient deficiencies such as vitamins, minerals, amino acids, omega 3 and carbohydrates were related to mental health illness such as depression, bipolar diseases, etc. Thus, dietary supplements usually help to alleviate mental illness symptoms [51]. Moreover, a study among 84 patients with PTSD showed that 62.7% had vitamin D insufficiency and 25.4% had vitamin deficiency [52]. Interestingly, consuming micronutrients post-disaster including earthquakes, floods and massacres showed that micronutrient supplements reduced the risk of PTSD from 75 to 15% [53]. However, one study using Twitter Sentiment Analysis concluded an individuals with mental health disorders and taking a supplement showed more negative emotions in their tweets [54]. Unfortunately, this study did not measure the level of micronutrients consumed by the respondents; thus, the cause and effect between hypervigilance and consuming dietary supplements could not be concluded. However, it might be possible that respondents with hypervigilance symptoms consumed dietary supplements to reduce the risk of SARS-CoV-2. In addition, a study among 610 Indonesian mothers concluded no association occurred between consuming supplements presenting mental health problems during the first year of the COVID-19 pandemic [8].

Moreover, respondents at younger ages were more likely to present avoidance symptoms as they were 2.439-times more likely to have avoidance symptoms (95% CI 1.339 to 4.443, p = 0.004). This study supports the finding among 111 children 3 to 8 years old and their young mothers in USA reporting 26% of the children experienced traumatic stress symptoms including hypervigilance, avoidance and intrusion as a stress response to the COVID-19 pandemic [55]. Similar findings were also reported among 610 Indonesian mothers, as younger and older mothers were more likely to develop mental health problems, compared with middle age mothers [8]. Moreover, a study among 3370 trauma-exposed adolescents from low and middle-income countries also concluded that younger age respondents exhibited a greater risk to develop post-traumatic stress events, even though they were not COVID-related events [56]. Remarkably, avoidance symptoms were usually observed to be higher among younger adults with inhibited temperament [57], which unfortunately was not investigated in this study.

In addition, higher education was a negative predictor for avoidance symptoms, as respondents graduating from high school were 0.546 less likely to have these symptoms (95% CI 0.323 to 0.921, p = 0.023) compared with respondents obtaining a diploma or higher levels. This finding differed from a study involving 2,244,193 Swedish adults concluding patients with PTSD were less likely to achieve good performance in education [58]. Additionally, a meta-analysis study involving 52 studies on PTSD and earthquakes found that respondents with lower education level exhibited a greater risk to develop PTSD [59]. This difference might have been caused because people with lower education levels did not have sufficient knowledge of COVID-19; and thus, were unafraid of or lacked stress regarding the risk. Moreover, our results showed that sociodemographic factors were not significantly related to somatic symptoms and re-experiencing symptoms from COVID-19 related psychological distress. This finding contrasted that of a study among 610 Indonesian mothers during the first year of the COVID-19 pandemic concluding no significant association existed between education level and mental health problems [8].

Age also might have contributed to societal anxiety during the early Omicron variant COVID-19 pandemic in Indonesia, as young adults were more likely to develop societal anxiety compared with middle-aged adults (10.94 ± 2.56 vs 10.17 ± 2.61, p = 0.011). This might have been caused by the higher mobility of young adults compared with middle-aged adults, as during the Omicron wave, social restrictions were more relaxed. This mobility caused younger people to exhibit a greater risk of contracting SARS-CoV-2 and spreading the disease, as reported by Rumain *et al.* [60]. In addition, we could not find other sociodemographic factors as predictors for societal influence including social distancing, social desirability and social information. It might have been caused during the early Omicron period when restrictions were relaxed after significantly loosened Delta variant cases and high coverage of COVID-19 vaccination were noted [61].

During the COVID-19 outbreak, increasing in mental health disorders was unavoidable. However, the services of mental health were disrupted, such as the re-assignment of healthcare professionals, access to healthcare facilities, face mask policy might interfere with the psychiatrist examination, etc [62,63]. Thus, to eliminate this problem, internet-based cognitive behavior therapy (iCBT) was developed. It showed high effectiveness to treat depression, anxiety, mood disorders and insomnia. A combination of iCBT and psychopharmacology showed higher effectiveness to treat mental health problems compared with a single method. It was also comparable with face-to-face CBT [64–66]. A study involving 84 Hongkong university students showed a reduction of depression, anxiety and stress symptoms after completion of eight modules of iCBT [67], and the Markov model analysis concluded that iCBT was cost-saving and effective for alleviating mild anxiety [68]. Even though CBT has been used widely in Indonesia, there was no report on iCBT, especially during the COVID-19 pandemic. However, several online applications, websites, and telemedicine have been developed for screening and counseling for mental health cases [69–71].

Despite using an online questionnaire that might cause limited access to the remote respondents, instability/unfamiliarity of internet access and inability to explore the occurrence of COVID-19 burnout which might affect many individuals, our study supported the importance of controlling mental health issues after experiencing traumatic COVID-19 cases. Sociodemographic factors might necessarily be considered for more effective measurement strategies to support mental health needs during catastrophic events. Moreover, randomized sampling is required to draw complete pictures of COVID-19 related psychological distress in Java Island or the broader region.

## Conclusion

The COVID-19 pandemic hits every aspect of human life, including social life and psychology. Our study shows the incidence of COVID-19-related psychological distress and social anxiety were still found in the early phase of the Omicron variant, especially among young adults. Since the psychological problems might affect the health condition and *vice versa*, understanding the COVID-19 psychological distress, the societal impact of the pandemic, and its determinant factors are vital for a better understanding of the vulnerability's variations during the COVID-19 outbreak, thus can be beneficial to deal with future catastrophic events, especially in a diverse country like Indonesia.

**Summary points**

- This study showed that almost 50% of respondents faced psychological distress during the early phase of the Omicron variant, especially concerning hypervigilance and avoidance.
- Several sociodemographic factors might have contributed to the incidence of psychological distress including age, comorbidities, younger age and education level.
- During the early stage of the Omicron wave, anxiety symptoms were detected, and their incidence correlated with age.
- Young adults were at risk for developing COVID-related psychological distress, which might require more attention from the stakeholders.
