# Supplementary material for: Societal influence and psychological distress among Indonesian adults in Java on the early Omicron wave of COVID-19
Source: Future Sci OA. 2023 Aug 29;9(10):FSO894. doi: 10.2144/fsoa-2023-0104 (PMC10518838; doi:10.2144/fsoa-2023-0104)
Supplement: Supplementary file 1 [file fsoa-09-894-s1.docx]

DRPST Questionnaire

| Measures | Items | Response scale |
| --- | --- | --- |
| COVID-related psychological distress (DRPST ^1^): In recent one month, do you have the following symptoms persisted more than one week due to COVID-19? | Item 1: Hypervigilance or difficulty to be relaxed, even without information about COVID-19. | 1 = not at all, 2 = minimal, 3 = moderate, 4 = predominant, 5 = extreme |
|  | Item 2: Emergence of somatic symptoms (e.g. palpitation, tremor, sweating, or muscle rigidity) when hearing about COVID-19 | 1 = not at all, 2 = minimal, 3 = moderate, 4 = predominant, 5 = extreme |
|  | Item 3: Efforts to avoid activities, places, people or information that arouse recollections of the COVID-19. | 1 = not at all, 2 = minimal, 3 = moderate, 4 = predominant, 5 = extreme |
|  | Item 4: Acting or feeling as if the trauma were recurring (re-experience), and feel distressed. | 1 = not at all, 2 = minimal, 3 = moderate, 4 = predominant, 5 = extreme |

SISQ Questionnaire

| No | How was COVID-19 impacted your society | Never 0% | Rarely 10% | Sometimes 30% | Often 60% |
| --- | --- | --- | --- | --- | --- |
| 1 | I constantly check for latest pandemic news updates via television, computer or phone |  |  |  |  |
| 2 | I avoid communication with or encountering strangers. |  |  |  |  |
| 3 | I avoid close or personal contact with family members and/or people I am close to |  |  |  |  |
| 4 | I worry about the pandemic affecting my work |  |  |  |  |
| 5 | I feel anxious or fearful due to the pandemic |  |  |  |  |
| 6 | I avoid going out, especially if I should require public transport |  |  |  |  |
| 7 | I continuously seek out information regarding COVID-19. |  |  |  |  |
| 8 | I believe that self-health management is helpful in controlling the spread of COVID-19 |  |  |  |  |
| 9 | I have faith in our current government’s epidemic response and risk management |  |  |  |  |
| 10 | I am bothered by social distancing during this period of epidemic response |  |  |  |  |
| 11 | I comply with the government’s implementations of epidemic response in the community |  |  |  |  |
| 12 | I am worried about COVID-19 and its impacts on our society, politics and economy |  |  |  |  |
| 13 | I reduce eating out |  |  |  |  |

Social distance: Q2, 3, 6, 13

Social Anxiety: Q4, 5, 10, 12

Social Desirability: Q8, 9, 11

Social Information: Q1, 7
